# Self-Medication Practices among Parents in Italy

**DOI:** 10.1155/2015/580650

**Published:** 2015-01-20

**Authors:** Luca Garofalo, Gabriella Di Giuseppe, Italo F. Angelillo

**Affiliations:** Department of Experimental Medicine, Second University of Naples, Naples, Italy

## Abstract

The aims of this cross-sectional survey were to document the prevalence, the determinants, and the reasons of oral medication use without the prescription of a physician among a random sample of 672 parents of students attending randomly selected public schools in Italy. A total of 69.2% practiced self-medication at least once. The odds of having performed a self-medication were higher in females, in younger population, and in those who have had a health problem in the preceding year and were lower in respondents with a middle or lower school level of education. Among those reporting experience of self-medication, 53.4% have practiced at least once in the last year and this was more likely for those who have had a health problem. Nonsteroidal anti-inflammatory drugs were more frequently used without a prescription in the last year. Two-thirds inappropriately self-medicated in the last year at least once. Of those who did not report a self-medication, 13.1% were willing to practice it. Females were more willing and those with a secondary school level of education less willing to practice self-medication. The frequency of oral self-medication was quite high and in most cases inappropriate with a potential impact on the health status and educative programs are needed.

## 1. Introduction

Self-medication is still an important public health problem throughout the world, since it is a fairly common practice. Unjustified and inappropriate self-medication results in wastage of healthcare resources and increases resistance of pathogens, drug-drug interactions, and adverse drug reactions leading to hospital admissions [1–4]. Sociocultural and socioeconomic characteristics, the previous experience with a symptom or disease, the attitude toward a disease, the way in which healthcare is funded or reimbursed, the increased potential to manage illnesses through self-care, and the availability of medicinal products have been quoted as explanatory factors of the self-medication [5–7].

In the past several years, self-medication has been studied in many areas and several articles have provided the prevalence among healthcare services attendants [4, 8–10], general population of adults [11–15] and adolescents [16–18], university students [19–22], and individuals with different health problems [23–26]. To our knowledge, no information is available regarding the self-medication prevalence in the community in Italy. Therefore, it is important to have data on self-medication in this area so that future interventions can be planned. Hence, the objectives of the present survey carried out in the general population in Italy were to document the prevalence of oral medication use without the prescription of a physician and to investigate the determinants and reasons that influence such practice.

## 2. Materials and Methods

From October through December 2012, a cross-sectional epidemiological study was conducted in the cities of Naples and Salerno, Italy. The study participants were a random sample of 989 parents of children aged 3–18 years within randomly selected classes in randomly selected public schools.

The manager of each selected school provided permission to conduct the study. After the approval, each child in the randomly selected classes at a participating school received an envelope from the research team addressed to the parents including an information sheet, an informed consent form, a copy of the questionnaire, and a self-addressed envelope for returning to the research team only the questionnaire. The predefined information sheet included the purpose and the procedure of the study, that participant privacy and confidentiality would be protected since no names or other personally identifying information were recorded, and asking only one parent to participate. The information regarding the voluntary nature of participation and the anonymity of the responses was also printed at the beginning of the questionnaire. Written informed consent was obtained from all the respondents and returned to the school manager. The interviewees did not receive any incentive to participate in the study.

The sample size necessary to reach the study objectives was estimated considering a 95% confidence interval, a 5% margin of error, and an expected 50% of subjects who have practiced an oral self-medication. With an allowance of 50% for nonresponse, the total sample size was estimated at 770 subjects.

The study team designed the questionnaire and conducted a preliminary pilot test on 40 subjects in order to test items' understandability and content validity. Data collected from these participants were used to make final refinements to the instrument. The questionnaire was reused on the same subjects to collect the same data a month later. The internal consistency and the reproducibility were assessed, respectively, by Cronbach's *α* and Cohen's kappa. The results of the pilot study showed a good consistency with *α* values higher than 0.9 and in relation to the reproducibility of each item, values were 0.8 or higher. Each enrolled participant filled in the questionnaire that had four sections. In the first section, there was included data on the basic demographics (gender, age, the highest attained education qualification, marital status, number of sons, and employment status), self-reported medical conditions, and self-rated health using a ten-point Likert-type scale ranging from poor (1 point) to excellent (10 points). In the second section, the participants were asked about the personal access to the healthcare services in the previous twelve months. In the third section, the respondents were asked whether they used an oral medication without the prescription of a physician. For those who reported a self-medication in the previous twelve months, information was asked regarding their medical history and the drugs/drug groups used for self-medication, the dosage and the duration of medication, and also the reasons for self-medication. For those who did not report a self-medication, they were asked about their willingness to practice medication without the prescription of a physician. In the last section, questions about the main source of and the needs of additional information regarding the use of medicines were collected.

Assessment of appropriateness of the self-medication for individual subjects was conducted using a tool adapted from the validated instrument Medication Appropriateness Index (MAI) [27].

Ethical approval was obtained from the Ethical Committee of the Second University of Naples which reviewed the proposal, questionnaire, and consent form before providing clearance.

## 3. Statistical Analysis

The statistical analysis has been completed in two stages. Initially, a series of Student's* t*-test for independent samples to assess differences between means and chi-square test to assess categorical differences were conducted to identify potential candidate variables for the logistic regression models. Following this stage, those variables achieving in the bivariate analysis a *P* value ≤ 0.25 were included in the multivariate logistic regression models, using backward stepwise regression of variables, to examine the effects of each independent variable on the different outcomes of interest. The criterion for entering and exiting the variables in the model was, respectively, being of *P* value > 0.2 and *P* value < 0.4. Four multivariate logistic regression models have been constructed: having used at least once some form of oral self-medication (Model 1); having used at least once some form of oral self-medication in the twelve-month period preceding the study (Model 2); appropriateness of oral self-medication (Model 3); and willingness to practice medication without the prescription of a physician (Model 4). The following characteristics of the respondent were included in all models: gender (male = 0; female = 1), age (continuous, in years), educational level (three categories: primary school or lower = 1; secondary school = 2; college degree or higher = 3), occupation (unemployed = 0; employed = 1), number of sons (three categories: one = 1; two = 2; three or more = 3), suffering of at least one chronic disease (cardiovascular diseases, diabetes, obesity, and hypertension) (no = 0; yes = 1), self-perceived health status (continuous), having personal health problem in the preceding twelve months (no = 0; yes = 1), visiting the physician in the preceding twelve months (no = 0; yes = 1), physicians as source of information about medicines (no = 0; yes = 1), and need of additional information about medicines (no = 0; yes = 1).

The results of the logistic regression models are presented as adjusted odds ratios (ORs) along with their 95% confidence intervals (CIs). A probability level of *P* ≤ 0.05 based on two-sided statistical tests was considered as denoting statistical significance. Data was analyzed using Stata version 10.1 software package [28].

## 4. Results

Out of 989 randomly selected people, in total, 672 participated for a response rate of 67.9%. The principal characteristics of the parents who filled the survey and their relationship on having some form of self-medication are detailed in Table 1. Approximately two-thirds were females; their mean age was 45.5 years and the vast majority had a secondary education level or higher; one-third were unemployed, the vast majority had more than one son; and three-quarters reported a personal history of chronic disease. Only 12.8% respondents rated their health as being very good, more than half reported never having a personal health problem in the preceding twelve months, and two-thirds reported that they had visited their doctor within the last twelve months.

Of the 672 participants, 465 (69.2%) stated that they had used an oral medication without the prescription of a physician at least once in their lives. The odds of having performed at least once some form of oral self-medication were higher in females (OR = 1.52; 95% CI 1.03–2.23), in those younger (OR = 0.96; 95% CI 0.93–0.99), and in individuals who have had a personal health problem in the preceding twelve months (OR = 1.64; 95% CI 1.15–2.34), whereas the odds were lower in respondents with a middle or lower school level of education (OR = 0.24; 95% CI 0.14–0.41) compared with those with a college degree or higher (Model 1 in Table 2). Among participants reporting an experience of oral self-medication, 334 (71.8%) reported having done so at least once in the 12-month period preceding the study. The likelihood of having ever performed a self-medication in the last year was more likely for individuals who have had a personal health problem in the preceding twelve months (OR = 1.65; 95% CI 1.09–2.48) (Model 2 in Table 2). The analysis of the drugs/drug groups used for oral self-medication by the 334 respondents in the 12-month period preceding the study showed that a total of 560 episodes were reported and the nonsteroidal anti-inflammatory drugs (NSAIDs) (83.5%) were most commonly used, whereas less frequently antibiotics (26.7%), antacids (4.2%), and corticosteroids (3.4%) have been used.

Participants engaged in self-medication most frequently because they felt that the illness was too mild and they did not require the services of a doctor (84.1%); other reasons were that they used an old prescribed medication (32.9%) and that they were prompted by a pharmacist (29%). A total of 65.6% respondents inappropriately self-medicated in the 12-month period at least once. No variables were identified as significantly predictive of this outcome in the multivariate logistic regression model (Model 3 in Table 2).

Of the 207 participants who neither reported an oral self-medication, 13% were willing to practice medication without the prescription of a physician. A multiple logistic regression model was developed with several variables being independent predictors of intention to practice self-medication. Females (OR = 3.4; 95% CI 1.15–10.1) were willing to practice self-medication, whereas those having a secondary school level of education (OR = 0.34; 95% CI 0.12–0.94) were less willing compared with those of higher education (Model 4 in Table 2). In the self-medication willingness group, an emergency (44.4%) and having a mild illness (40.7%) were motivating factors in their decision, whereas, among those who stated that they would not consider self-medication, the most common reasons were that they trust in the physician (65%) and that they were concerned about the risk of side effects (42.2%).

For this sample, the main source of information on the use of medicines is the physician (70.5%), followed by information leaflet (63.5%) and pharmacist (39.9%), but they are also influenced by the internet (16.4%) and the media (4.6%). One-third (33.9%) of the respondents reported the need to obtain more information about the use of medicines.

## 5. Discussion

This observational survey provided the opportunity to examine the practices of oral self-medication among a large community in Italy and the findings represent a detailed characterization of the prevalence of this phenomenon and how different factors influence self-care, adding to the limited information and contributing to the existing literature.

The results of the survey indicate that oral self-medication is a common experience in the general population. According to similar studies, the prevalence of self-medication was lower than the values of 87% among Palestinian population [15] and of 75% in consumers of community pharmacies in Chile [11]. A study in Nigeria showed that 67.7% of the mothers treated their infants with colic without consulting a doctor [29]. The prevalence observed was higher than that in the general population in Jordan with a self-medication reported by 42.5% of cases interviewed [12], by 53.5%, although during the last 30 days, among older residents in Mexico [16], and by 18.1% of the Spanish adult population [30]. Moreover, surveys regarding the frequency of self-medication of antibiotics showed a huge variation with values of 7.8% among the Chinese in Hong Kong [31], 23% in the population of twelve countries in Europe [32], and 32.7% in the general population in the same area of the present study [33]. Widespread use and misuse of antibiotics in the community are of particular concern since antimicrobial-resistant bacteria are common in communities with frequent nonprescription use and an excessive and inappropriate use determine an increased risk of side effects and a significant economic impact [34, 35]. Efforts to contain unnecessary antibiotic use are necessary in order to restrict the spread of antimicrobial-resistant organisms.

The top drugs self-used by the respondents were the NSAIDs and this is in accordance with the already mentioned studies conducted in Chile [11, 13]. Motivating factors for self-medications were also investigated. The vast majority of the interviewees performed a self-medication because they felt that the illness was nonserious and they did not require the services of a doctor. This is consistent with previous studies where the most common reason was that the ailments were too minor to see a doctor [11, 12, 15]. It is interesting to observe that approximately one-fourth of those engaged in self-medication were prompted by pharmacists. Although our regulations do not allow pharmacists to prescribe drugs, this may be explained by the fact they are more easily accessible and the service is faster.

In this population, the results of the multivariate logistic regression analysis demonstrated that several factors emerged as being significantly associated with the different outcomes of interest. Self-medication practice was strongly influenced by sociodemographic indicators such as gender, age, and educational level. Indeed, participants seeking self-treatment were predominantly female, younger, and more educated—a proxy for socioeconomic status. Differences in the frequency of self-medication by gender were well documented in the literature, with older women more frequently self-medicated. In Spain, the women had a higher probability of indulging in self-medication [30]; in Brazil, among first- and last-year students enrolled in healthcare and nonhealthcare programs; being male was a protection factor against self-medication [19] and women with endocrinopathies and metabolic diseases used more dietary supplements [24]. In adults in an urban setting in Jordan, those who had taken at least one medication without prescription were more likely to be younger [12] and in the survey in Hong Kong respondents aged below 40 were more likely to buy nonprescribed antibiotics [31]. Among the sample in Spain, a greater probability of self-medication was observed among those with a higher level of education [30]. Moreover, it is interesting to observe that respondents with a personal health problem in the preceding twelve months were more likely to perform self-medication.

The results of this study should be interpreted keeping in mind some limitations. The primary limitation is the design that was used, in the sense that cross-sectional studies do not permit ascertaining causal inferences for the effects of the dependent variables on the outcomes. Second, this study utilized a sample of parents of schooled children from one Italian region and concern about generalizability of the results may arise. This population, compared to the general population, probably underestimates people older than 50 years and excludes those without sons. Third, this study utilized a sample of self-identified parent, which is vulnerable to selection bias as those in the family with strong interest in this topic might have preferentially responded. However, any significant difference within the family was not expected. Fourth, the participant self-reported the information, with the inability of the researchers to validate with objective measures the answers and some may overreport socially desirable attitudes and/or behaviors or underreport socially undesirable attitudes and/or behaviors. The absence of identifying data on the questionnaire sheets would tend to minimize such bias. Fifth, as in all survey research, those who did not return the survey may have beliefs and behaviors that differ from those that responded. However, in spite of these limitations, the main strength of this study is the use of a large and properly selected sample.

In conclusion, the frequency of self-medication was quite high within this community, mainly in female, younger, and more educated groups, and was in most cases inappropriate with a potential impact on the health status and, therefore, educative and preventive programs are needed.

## Figures and Tables

**Table 1:** Characteristics of study participants and self-medication practices.

Characteristics	Total		Self-medication practice				Last year self-medication practice				Last year appropriateness self-medication			
			Yes = 465 (69.2%)		No = 207 (30.8%)		Yes = 334 (71.8%)		No = 131 (28.2%)		Yes = 115 (34.4%)		No = 219 (65.6%)	
	*N*	%	*N*	%	*N*	%	*N*	%	*N*	%	*N*	%	*N*	%
Gender														
Male	182	27.1	112	24.1	70	33.8	85	25.5	27	20.6	23	20	62	28.3
Female	490	72.9	353	75.9	137	66.2	249	74.5	104	79.4	92	80	157	71.7
			*P* = 0.009				*P* = 0.27				*P* = 0.1			
Age	45.5 ± 6.7 (24–70)^*^		45 ± 6.5 (24–70)^*^		46.6 ± 7 (32–68)^*^		44.8 ± 6 (24–65)		45.5 ± 7.6 (30–70)		44.1 ± 6 (31–64)^*^		45.1 ± 6 (24–65)^*^	
<40	106	15.8	82	17.6	24	11.6	57	17.1	25	19.1	21	18.3	36	16.4
40–44	205	30.5	144	31	61	29.5	104	31.1	40	30.5	41	35.6	63	28.8
45–49	216	32.1	150	32.3	66	31.9	113	33.8	37	28.2	39	33.9	74	33.8
>49	145	21.6	89	19.1	56	27	60	18	29	22.2	14	12.2	46	21
			*P* = 0.003				*P* = 0.26				*P* = 0.18			
Educational level														
Primary school or lower	94	14	45	9.7	49	23.7	34	10.2	11	8.4	9	7.8	25	11.4
Secondary school	308	45.8	217	46.7	91	43	150	44.9	67	51.1	51	44.4	99	45.2
College degree or higher	270	40.2	203	43.6	67	32.3	150	44.9	53	40.5	55	47.8	95	43.4
			*P* ≤ 0.001				*P* = 0.47				*P* = 0.52			
Occupation														
Employed	436	64.9	311	66.9	125	60.4	228	68.3	83	63.4	75	65.2	153	69.9
Unemployed	236	35.1	154	33.1	82	39.6	106	31.7	48	36.6	40	34.8	66	30.1
				*P* = 0.1			*P* = 0.31				*P* = 0.39			
Number of sons														
1	96	14.3	67	14.4	29	14	48	14.4	19	14.5	15	13.1	33	15.1
2	395	58.8	274	58.9	121	58.5	198	59.3	76	58	71	61.7	127	58
≥3	181	26.9	124	26.7	57	27.5	88	26.3	36	27.5	29	25.2	59	26.9
			*P* = 0.97				*P* = 0.97				*P* = 0.79			
Self-perceived health status	7.7 ± 1.5 (1–10)^*^		7.7 ± 1.4 (2–10)^*^		7.6 ± 1.7 (1–10)^*^		7.7 ± 1.4 (2–10)^*^		7.7 ± 1.4 (4–10)^*^		7.9 ± 1.4 (2–10)^*^		7.5 ± 1.4 (2–10)^*^	
			*P* = 0.89				*P* = 0.87				*P* = 0.01			
Suffering of at least one chronic disease														
No	172	25.6	115	24.7	57	27.5	252	75.5	98	74.8	92	80	160	73.1
Yes	500	74.4	350	75.3	150	72.5	82	24.5	33	25.2	23	20	59	26.9
			*P* = 0.44				*P* = 0.89				*P* = 0.16			
Personal health problem in the preceding twelve months														
No	370	55.1	243	52.3	127	61.3	163	48.8	80	61.1	69	60	94	42.9
Yes	302	44.9	222	47.7	80	38.7	171	51.2	51	38.9	46	40	125	57.1
			*P* = 0.03				*P* = 0.02				*P* = 0.003			
Had visited the physician in the preceding twelve months														
No	176	26.2	123	26.5	53	25.6	81	24.2	42	32.1	36	31.3	45	20.5
Yes	496	73.8	342	73.5	154	74.4	253	75.8	89	67.9	79	68.7	174	79.5
			*P* = 0.82				*P* = 0.09				*P* = 0.03			
Physicians as source of information about medicines														
No	198	29.5	145	31.2	53	25.6	107	32	38	29	35	30.4	72	32.9
Yes	474	70.5	320	68.8	154	74.4	227	68	93	71	80	69.6	147	67.1
			*P* = 0.14				*P* = 0.53				*P* = 0.65			
Need of additional information about medicines														
No	228	33.9	302	65	142	68.6	215	64.4	87	66.4	83	72.2	132	60.3
Yes	444	66.1	163	35	65	31.4	119	35.6	44	33.6	32	27.8	87	39.7
			*P* = 0.36				*P* = 0.68				*P* = 0.03			

^*^Mean ± standard deviation (range).

**Table 2:** Logistic regression models for potential determinants of the different outcomes of interest.

	OR	95% CI	*P* value
Model 1. Have taken at least once some form of self-medication (*n* = 672)			
Educational level			
Primary school or lower	0.24	0.14–0.41	<0.001
Secondary school	0.69	0.47–1.01	0.051
College degree or higher	1.0^*^	—	—
Age	0.96	0.93–0.99	0.003
Personal health problem in the preceding twelve months	1.64	1.15–2.34	0.006
Gender	1.52	1.03–2.23	0.03
Physician as source of information about medicines	0.69	0.47–1.02	0.06
Model 2: Self-medication in the twelve-month period preceding the study (*n* = 465)			
Health problem in the preceding twelve months	1.65	1.09–2.48	0.02
Model 3: Appropriateness of oral self-medication (*n* = 334)			
Gender	1.74	0.99–3.05	0.051
Need of additional information about medicines	0.63	0.38–1.04	0.07
Self-perceived health status	1.17	0.38–1.07	0.09
Personal health problem in the preceding twelve months	0.64	0.8–5.41	0.14
Had visited the physician in the preceding twelve months	0.74	0.42–1.31	0.31
Model 4: Willing to practice medication without the prescription of a physician (*n* = 207)			
Gender	3.4	1.15–10.1	0.03
Educational level			
Primary school or lower	0.19	0.03–1.13	0.07
Secondary school	0.34	0.12–0.94	0.04
College degree or higher	1.0^*^	—	—
Occupation	2.89	0.81–10.24	0.1
Personal health problem in the preceding twelve months	2.07	0.8–5.41	0.14
Had visited the physician in the preceding twelve months	2.79	0.7–11.08	0.14
Age	1.05	0.98–1.12	0.19

^*^Reference category.

## References

[1] Olivier P., Bertrand L., Tubery M., Lauque D., Montastruc J.-L., Lapeyre-Mestre M. (2009). Hospitalizations because of adverse drug reactions in elderly patients admitted through the emergency department: a prospective survey. *Drugs & Aging*.

[2] Lewinski D., Wind S., Belgardt C., Plate V., Behles C., Schweim H. G. (2010). Prevalence and safety-relevance of drug-related problems in German community pharmacies. *Pharmacoepidemiology and Drug Safety*.

[3] Eickhoff C., Hämmerlein A., Griese N., Schulz M. (2012). Nature and frequency of drug-related problems in self-medication (over-the-counter drugs) in daily community pharmacy practice in Germany. *Pharmacoepidemiology and Drug Safety*.

[4] Asseray N., Ballereau F., Trombert-Paviot B. (2013). Frequency and severity of adverse drug reactions due to self-medication: a cross-sectional multicentre survey in emergency departments. *Drug Safety*.

[5] Hughes C. M., McElnay J. C., Fleming G. F. (2001). Benefits and risks of self medication. *Drug Safety*.

[6] Martins A. P., da Costa Miranda A., Mendes Z., Soares M. A., Ferreira P., Nogueira A. (2002). Self-medication in a Portuguese urban population: a prevalence study. *Pharmacoepidemiology and Drug Safety*.

[7] Al-Azzam S. I., Al-Husein B. A., Alzoubi F., Masadeh M. M., Al-Horani M. A. (2007). Self-medication with antibiotics in Jordanian population. *International Journal of Occupational Medicine and Environmental Health*.

[8] Ilhan M. N., Durukan E., Ilhan S. Ö., Aksakal F. N., Özkan S., Bumin M. A. (2009). Self-medication with antibiotics: Questionnaire survey among primary care center attendants. *Pharmacoepidemiology and Drug Safety*.

[9] Skliros E., Merkouris P., Papazafiropoulou A. (2010). Self-medication with antibiotics in rural population in Greece: a cross-sectional multicenter study. *BMC Family Practice*.

[10] Roulet L., Asseray N., Foucher N., Potel G., Lapeyre-Mestre M., Ballereau F. (2013). A questionnaire to document self-medication history in adult patients visiting emergency departments. *Pharmacoepidemiology and Drug Safety*.

[11] Fuentes Albarrán K., Villa Zapata L. (2008). Analysis and quantification of self-medication patterns of customers in community pharmacies in southern Chile. *Pharmacy World and Science*.

[12] Yousef A.-M. M., Al-Bakri A. G., Bustanji Y., Wazaify M. (2008). Self-medication patterns in Amman, Jordan. *Pharmacy World and Science*.

[13] Balbuena F. R., Aranda A. B., Figueras A. (2009). Self-medication in older urban Mexicans: an observational, descriptive, cross-sectional study. *Drugs & Aging*.

[14] Hassali M. A., Shafie A. A., Al-Qazaz H., Tambyappa J., Palaian S., Hariraj V. (2011). Self-medication practices among adult population attending community pharmacies in Malaysia: an exploratory study. *International Journal of Clinical Pharmacy*.

[15] Al-Ramahi R. (2013). Patterns and attitudes of self-medication practices and possible role of community pharmacists in Palestine. *International Journal of Clinical Pharmacology and Therapeutics*.

[16] Ylinen S., Hämeen-Anttila K., Sepponen K., Lindblad A. K., Ahonen R. (2010). The use of prescription medicines and self-medication among children—a population-based study in Finlandy. *Pharmacoepidemiology and Drug Safety*.

[17] Bertoldi A. D., Silveira M. P. T., Menezes A. M. B., Assunção M. C. F., Gonçalves H., Hallal P. C. (2012). Tracking of medicine use and self-medication from infancy to adolescence: 1993 Pelotas (Brazil) birth cohort study. *Journal of Adolescent Health*.

[18] Opaleye E. S., Noto A. R., Sanchez Z. M. (2013). Nonprescribed use of tranquilizers or sedatives by adolescents: a Brazilian national survey. *BMC Public Health*.

[19] Corrêa da Silva M. G., Soares M. C. F., Muccillo-Baisch A. L. (2012). Self-medication in university students from the city of Rio Grande, Brazil. *BMC Public Health*.

[20] Donkor E. S., Tetteh-Quarcoo P. B., Nartey P., Agyeman I. O. (2012). Self-medication practices with antibiotics among tertiary level students in Accra, Ghana: a cross-sectional study. *International Journal of Environmental Research and Public Health*.

[21] Pan H., Cui B., Zhang D., Farrar J., Law F., Ba-Thein W. (2012). Prior knowledge, older age, and higher allowance are risk factors for self-medication with antibiotics among University students in Southern China. *PLoS ONE*.

[22] Kumar N., Kanchan T., Unnikrishnan B. (2013). Perceptions and practice of self-medication among medical students in coastal South India. *PLoS ONE*.

[23] Ayele K., Tesfa B., Abebe L., Tilahun T., Girma E. (2012). Self care behavior among patients with diabetes in Harari, Eastern Ethiopia: the health belief model perspective. *PLoS ONE*.

[24] Heller T., Müller N., Kloos C., Wolf G., Müller U. A. (2012). Self medication and use of dietary supplements in adult patients with endocrine and metabolic disorders. *Experimental and Clinical Endocrinology and Diabetes*.

[25] Rabin A. S., Kuchukhidze G., Sanikidze E., Kempker R. R., Blumberg H. M. (2013). Prescribed and self-medication use increase delays in diagnosis of tuberculosis in the country of Georgia. *International Journal of Tuberculosis and Lung Disease*.

[26] Mattila M., Boehm L., Burke S. (2013). Nonprescription medication use in patients with heart failure: assessment methods, utilization patterns, and discrepancies with medical records. *Journal of Cardiac Failure*.

[27] Hanlon J. T., Schmader K. E., Samsa G. P. (1992). A method for assessing drug therapy appropriateness. *Journal of Clinical Epidemiology*.

[28] Stata Corporation (2007). *Stata Reference Manual Release 10.1*.

[29] Oshikoya K. A., Senbanjo I. O., Njokanma O. F. (2009). Self-medication for infants with colic in Lagos, Nigeria. *BMC Pediatrics*.

[30] Carrasco-Garrido P., Jiménez-García R., Barrera V. H., Gil de Miguel A. (2008). Predictive factors of self-medicated drug use among the Spanish adult population. *Pharmacoepidemiology and Drug Safety*.

[31] Wun Y. T., Lam T. P., Lam K. F., Ho P. L., Yung W. H. R. (2013). The public's perspectives on antibiotic resistance and abuse among Chinese in Hong Kong. *Pharmacoepidemiology and Drug Safety*.

[32] Grigoryan L., Burgerhof J. G. M., Degener J. E. (2008). Determinants of self-medication with antibiotics in Europe: the impact of beliefs, country wealth and the healthcare system. *Journal of Antimicrobial Chemotherapy*.

[33] Napolitano F., Izzo M. T., Di Giuseppe G., Angelillo I. F. (2013). Public knowledge, attitudes, and experience regarding the use of antibiotics in Italy. *PLoS ONE*.

[34] Goossens H., Ferech M., Vander Stichele R., Elseviers M., ESAC Project Group (2005). Outpatient antibiotic use in Europe and association with resistance: a cross-national database study. *The Lancet*.

[35] Morgan D. J., Okeke I. N., Laxminarayan R., Perencevich E. N., Weisenberg S. (2011). Non-prescription antimicrobial use worldwide: a systematic review. *The Lancet Infectious Diseases*.

